# Curcumin increases breast cancer cell sensitivity to cisplatin by decreasing FEN1 expression

**DOI:** 10.18632/oncotarget.24109

**Published:** 2018-01-10

**Authors:** Jiao Zou, Linlin Zhu, Xiaomei Jiang, Yang Wang, Yue Wang, Xiangwei Wang, Bin Chen

**Affiliations:** ^1^ Department of Biochemistry and Molecular Biology, Third Military Medical University, Chongqing 400038, China; ^2^ Department of Urology, Shenzhen University General Hospital, Shenzhen 518060, Guangdong, China

**Keywords:** FEN1, curcumin, cisplatin resistance, breast cancer, ERK

## Abstract

Flap endonuclease 1 (FEN1) overexpression promotes breast cancer. We investigated the role of FEN1 in cisplatin resistance and the chemosensitizing effects of curcumin in breast cancer cells. We demonstrated that FEN1 overexpression promotes cisplatin resistance in breast cancer cells, and that FEN1 knockdown enhances cisplatin sensitivity. Curcumin down-regulated FEN1 expression in a dose-dependent manner. A combination of cisplatin and curcumin enhanced breast cancer cell sensitivity to cisplatin by down-regulating FEN1 expression *in vitro* and *in vivo*. Increased ERK phosphorylation contributed to cisplatin resistance and cisplatin-induced FEN1 overexpression in breast cancer cells. Inhibiting ERK phosphorylation stimulated the chemosensitizing effect of curcumin to cisplatin by targeting FEN1. These data reveal that FEN1 overexpression promotes cisplatin resistance, and suggest FEN1 could be a potential therapeutic target to relieve cisplatin resistance in breast cancer. We also demonstrated that curcumin sensitizes breast cancer cells to cisplatin through FEN1 down-regulation.

## INTRODUCTION

Cisplatin (cis-diammedichloroplatinum, DDP) is a powerful antineoplastic drug that treats solid tumors, including head and neck, lung, testis, ovary, and breast cancers [1, 2]. Cisplatin resistance frequently occurs in patients, which offsets the cisplatin-induced inhibited DNA repair. Structure-specific nuclease family members repair DNA damage by removing damaged bases, nucleotides, or various DNA intermediate structures [3]. Flap endonuclease 1 (FEN1) is a member of the family that promotes DNA replication and repair [4].

As a structure-specific nuclease, FEN1 stimulates Okazaki fragment maturation during DNA repair and its efficient removal of 5’-flaps during long-patch base excision repair [5]. FEN1 is also reported to possess the activities of 5’ end exonuclease and gap dependence endonuclease, which can promote apoptosis-induced DNA fragmentation in response to apoptotic stimuli [5, 6]. FEN1 maintains the stability of the genome that supports multiple DNA metabolic pathways.

FEN1 promotes cell proliferation [7], and its deficiency or overexpression can result in predisposition to cancer and the rapid development of tumors [8]. In breast cancer and prostate cancer, the overexpression of FEN1 is characterized as a possible biomarker for monitoring the progression of cancers and a potential target for therapy [9, 10]. In LN308 glioblastoma cells, FEN1 up-regulation is followed by cell resistance to chemotherapeutic drugs such as cisplatin, temozolomide, nimustine, and methyl methanesulfonate [11]. FEN1 overexpression can also cause resistance to many chemotherapeutic drugs in breast cancer [3].

Curcumin is a polyphenol extracted from turmeric root, and can induce tumor cell apoptosis and inhibit breast cancer tumor growth *in vitro* and *in vivo* [12–15]. Our previous study demonstrated that curcumin inhibits cell proliferation through the down-regulation of FEN1 expression in breast cancer cells [16]. We explored curcumin’s effect on cisplatin sensitivity and FEN1 expression.

## RESULTS

### Cisplatin sensitivity in MCF-7 and MCF-7DDP cells

**Figure 1:**
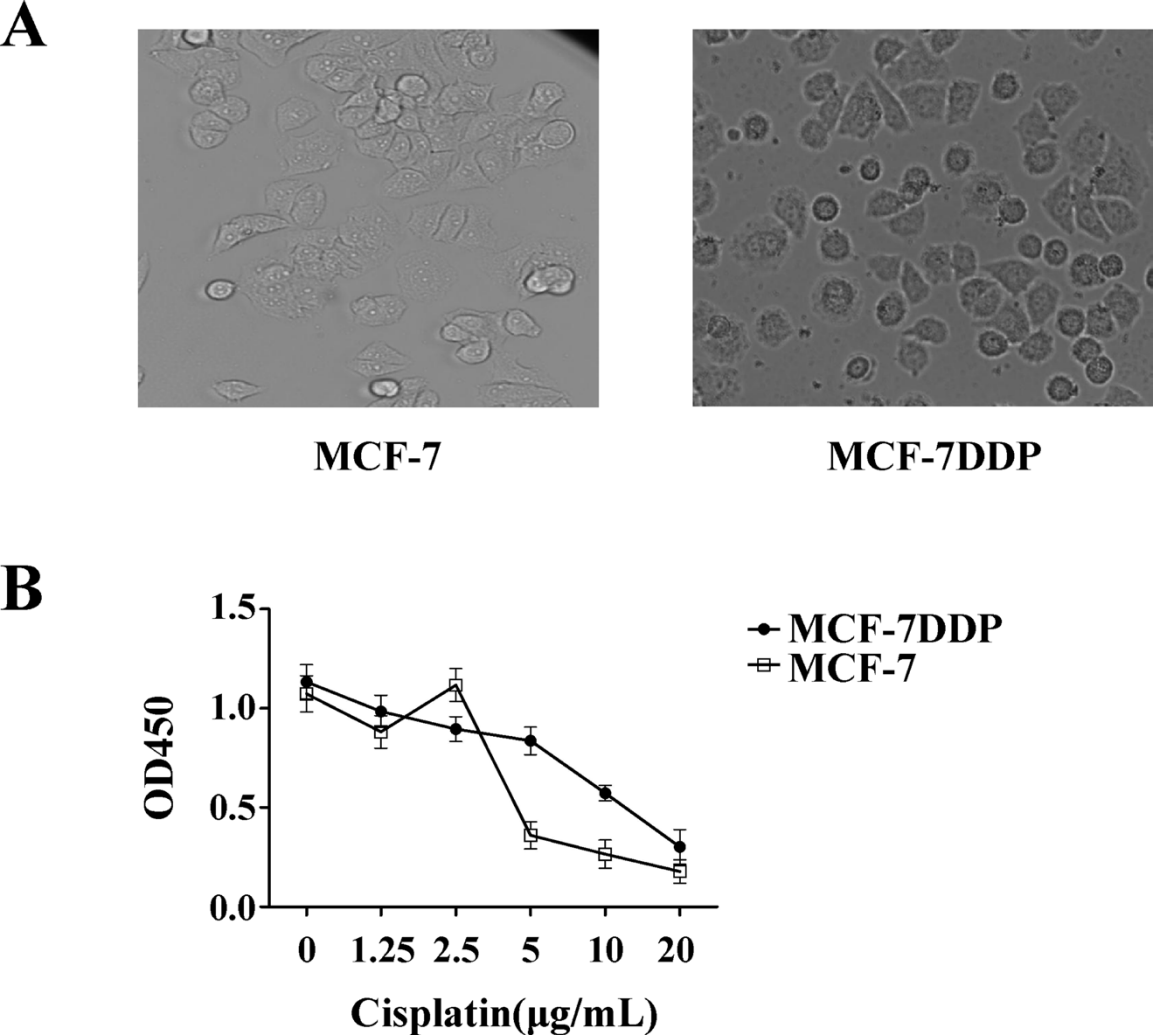
Cisplatin’s effect on breast cancer cell proliferation (**A**) Characterization of MCF-7DDP cells. MCF-7 and MCF-7DDP cells were treated with 5 μg/mL of cisplatin for 48 h, and the surviving cell numbers and cell morphology were observed by microscope. (**B**) MCF-7 and MCF-7DDP cells were treated with increasing concentrations of cisplatin for 48 h, and cell proliferation was determined by CCK-8 assay.

MCF-7 and cisplatin-resistant MCF-7DDP cells were treated with 5 μg/mL of cisplatin for 48 h, and the surviving cell numbers and cell morphology were observed with a microscope. Morphology differed between the two cell types, and there were more surviving MCF-7DDP cells than MCF-7 cells, as shown by micrographs (Figure 1A). This suggests that MCF-7DDP cells were resistant to 5 μg/mL cisplatin.

MCF-7 and MCF-7DDP cells were treated with increasing cisplatin concentrations for 48 h, and cisplatin’s effect on cell proliferation was detected using the CCK-8 assay (Figure 1B). Low cisplatin concentrations had no effect on MCF-7 and MCF-7DDP cell proliferation; high cisplatin concentrations inhibited MCF-7 and MCF-7DDP cell proliferation in a dose-dependent manner (*P* < 0.05). The IC50 value of cisplatin against MCF-7 and MCF-7DDP were 4 μg/mL and 15 μg/mL, respectively.

### FEN1 overexpression promotes cisplatin resistance in breast cancer cells

**Figure 2:**
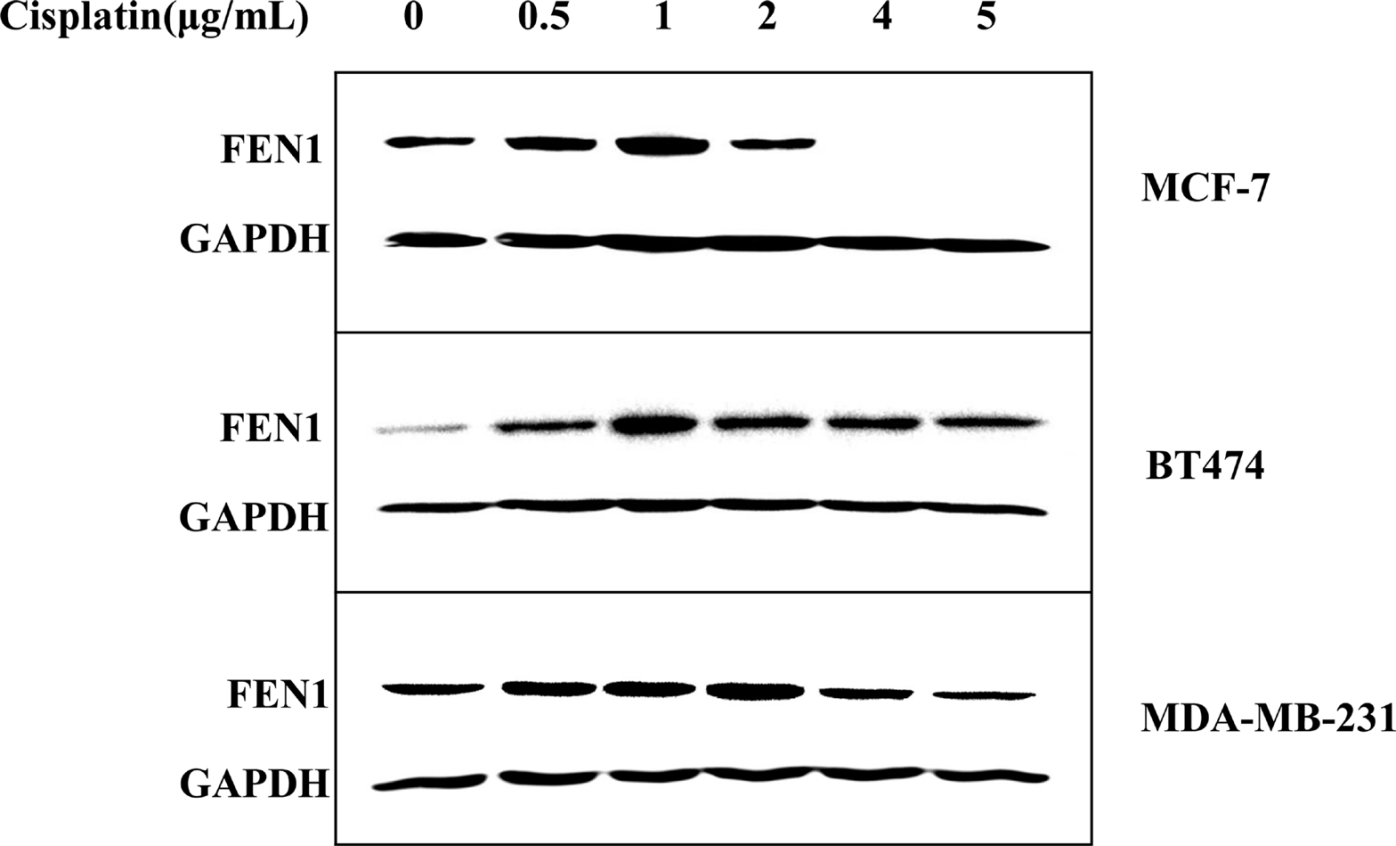
Cisplatin-induced up-regulation of FEN1 protein expression in breast cancer cells MCF-7, BT-474, and MDA-MB-231 cells were treated with increasing concentrations of cisplatin for 24 h, and FEN1 protein expression was analyzed by western blotting.

**Figure 3:**
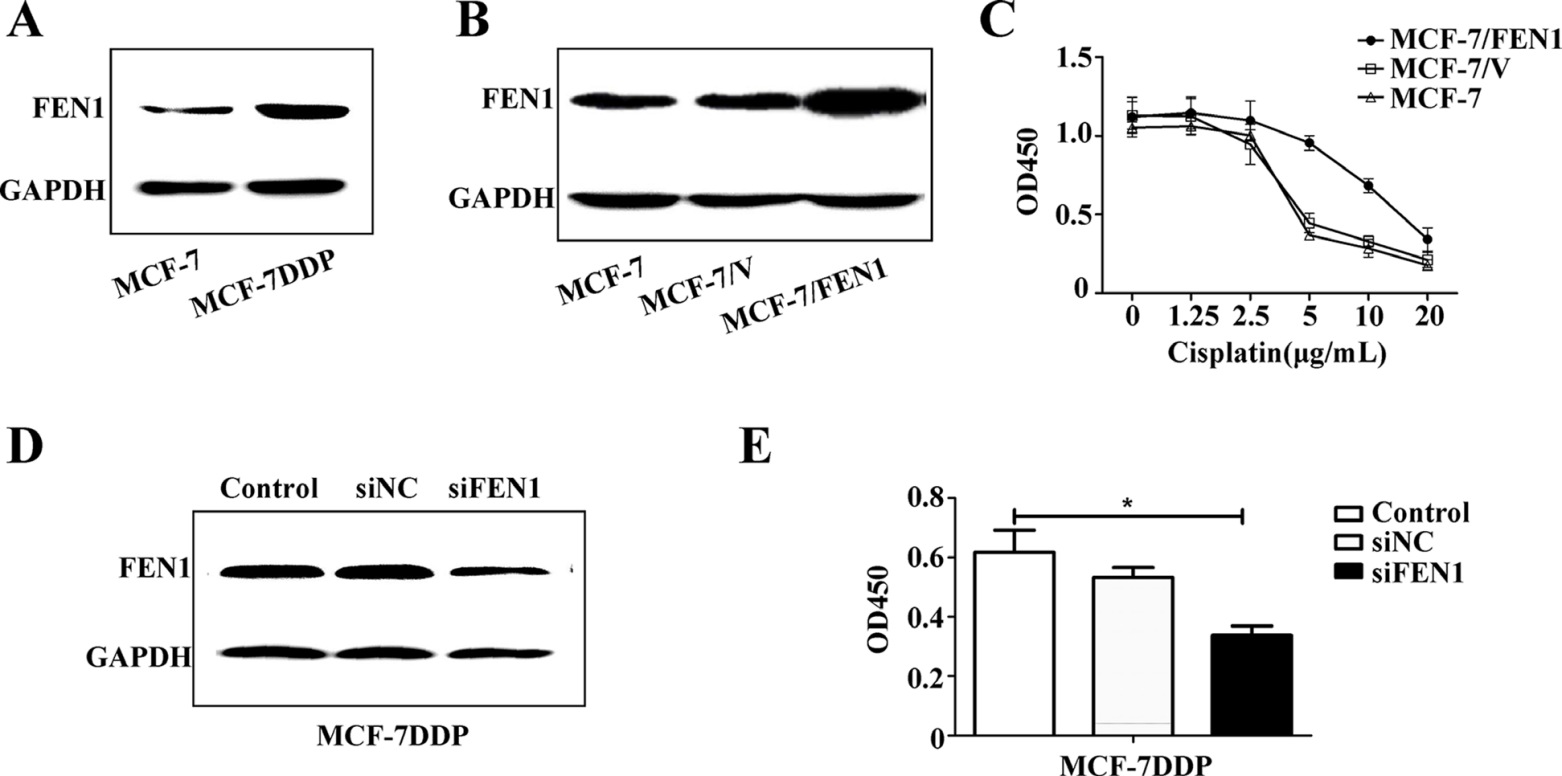
FEN1 overexpression promotes cisplatin resistance in breast cancer cells (**A**) Different protein levels of FEN1 in MCF-7 and MCF-7DDP cells. Lysates of MCF-7 and MCF-7DDP cells cultured in regular media were prepared and tested for FEN1 content by western blotting. (**B**) MCF-7 cells stably overexpressing FEN1 were screened by G418 for four weeks and identified by western blot. (**C**) MCF-7 cells stably overexpressing FEN1 or cells transfected with empty plasmid were treated with increasing concentrations of cisplatin for 48 h, and cell proliferation was determined by CCK-8 assay. (**D**) MCF-7DDP cells were transfected with FEN1 siRNA and its negative control siRNA (NC siRNA) for 48 h. Cells were collected and analyzed for FEN1 protein expression using western blotting. (**E**) The transfected MCF-7DDP cells were treated with or without 5 μg/mL cisplatin for 48 h and cell proliferation was analyzed by CCK-8 assay. ^*^P < 0.05.

To investigate FEN1 expression in cisplatin resistance, MCF-7, BT-474, and MDA-MB-231 breast cancer cell lines were treated with increasing cisplatin concentrations. FEN1 expression was analyzed by qPCR and western blot (Figure 2 and Supplementary Figure 1). Both mRNA and protein levels of FEN1 were up-regulated in a dose-dependent manner in three kinds of cells treated with low concentrations of cisplatin. FEN1 levels were suppressed in cells treated with high cisplatin concentrations, which may be related to the high cytotoxicity of cisplatin. FEN1 expression in MCF-7DDP cells was higher than in MCF-7 cells (Figure 3A, *P* < 0.05), indicating that FEN1 up-regulation was correlated with cisplatin resistance.

To further explore FEN1 overexpression in cisplatin resistance, MCF-7 cells stably overexpressing FEN1 were screened and identified (Figure 3B), and cisplatin sensitivity was detected (Figure 3C). Cisplatin sensitivity in MCF-7 cells stably overexpressing FEN1 was reduced compared with wild-type MCF-7 cells or MCF-7 cells transfected with empty plasmid. This suggests that FEN1 overexpression promotes cisplatin resistance in breast cancer cells.

To further confirm this conclusion, FEN1 gene expression in MCF-7DDP cells was silenced using RNAi, and changes in cell proliferation were analyzed (Figure 3D and 3E). Western blot analysis showed that siFEN1 transfection induced a FEN1 knockdown compared with the control transfection (Figure 3D). CCK-8 analysis showed that the proliferation of MCF-7DDP cells transfected with siFEN1 was reduced compared with control cells (Figure 3E). These data demonstrate that FEN1 overexpression stimulates cisplatin resistance, and that FEN1 down-regulation could enhance breast cancer cell sensitivity to cisplatin.

### Curcumin down-regulates FEN1 expression and inhibits human breast cancer cell proliferation

**Figure 4:**
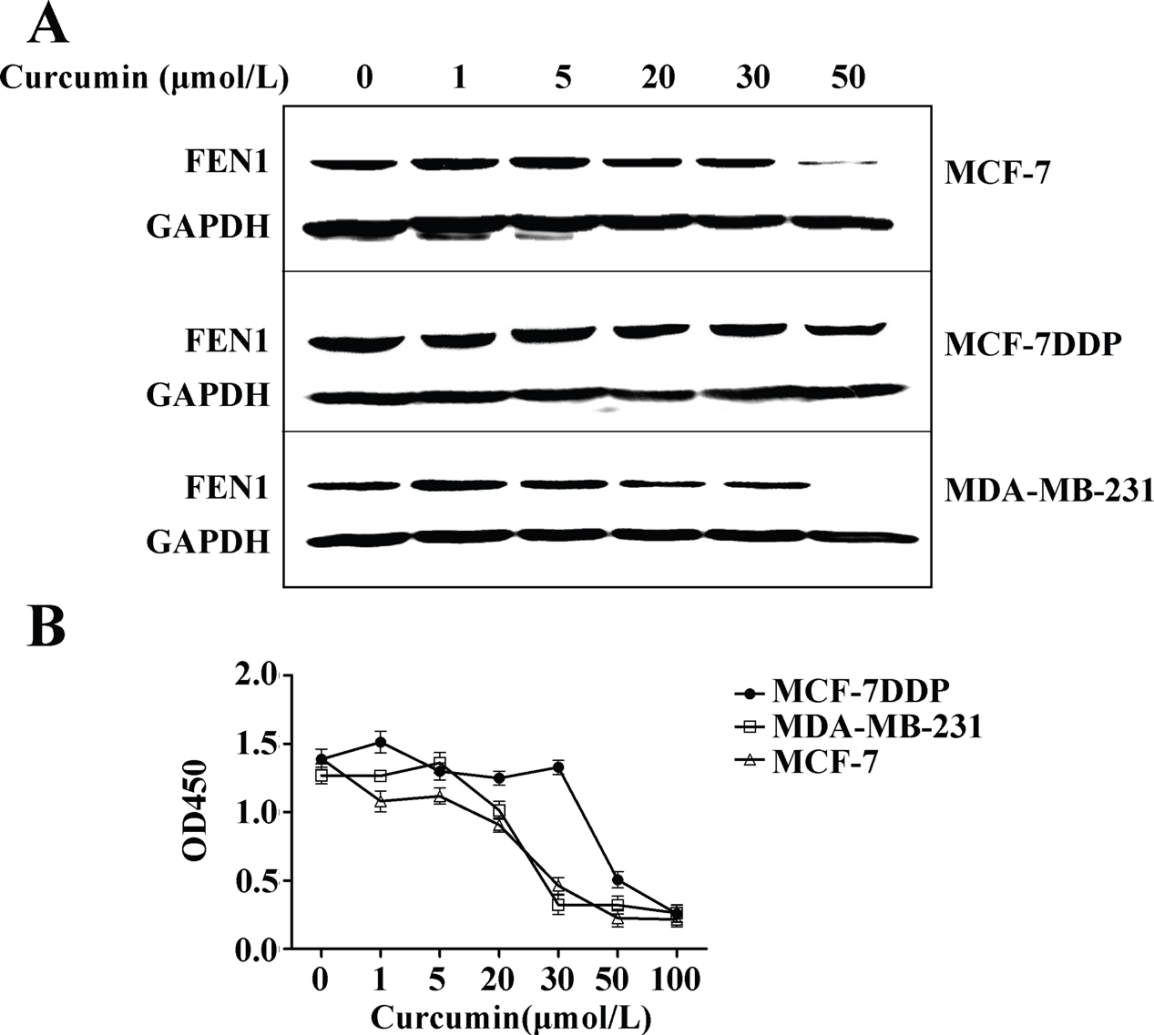
Curcumin’s effect on FEN1 expression and proliferation of breast cancer cells (**A**) MCF-7, MCF-7DDP and MDA-MB-231 cells were treated with increasing concentrations of curcumin for 24 h and then FEN1 protein expression was analyzed by western blotting. (**B**) MCF-7, MCF-7DDP, and MDA-MB-231 cells were treated with increasing concentrations of curcumin for 48 h, and cell proliferation was analyzed by CCK-8 assay.

MCF-7, MCF-7DDP, and MDA-MB-231 cells were treated with increasing curcumin concentrations, and the effect on FEN1 expression and cell proliferation were analyzed (Figure 4). FEN1 protein expression was decreased in all three kinds of cells in a dose-dependent manner (Figure 4A), and the proliferation of all three cells could be inhibited in a dose-dependent manner by curcumin (Figure 4B, *P* < 0.05). The IC50 value of curcumin against MCF-7, MCF-7DDP, and MDA-MB-231 was 25 μmol/L, 40 μmol/L, and 26 μmol/L, respectively. In accordance with our previous data [16], these results suggested that curcumin could inhibit breast cancer cell proliferation, including cisplatin-resistant cells through FEN1 down-regulation.

### The combination of cisplatin and curcumin enhances breast cancer cell sensitivity to cisplatin through FEN1 down-regulation

**Figure 5:**
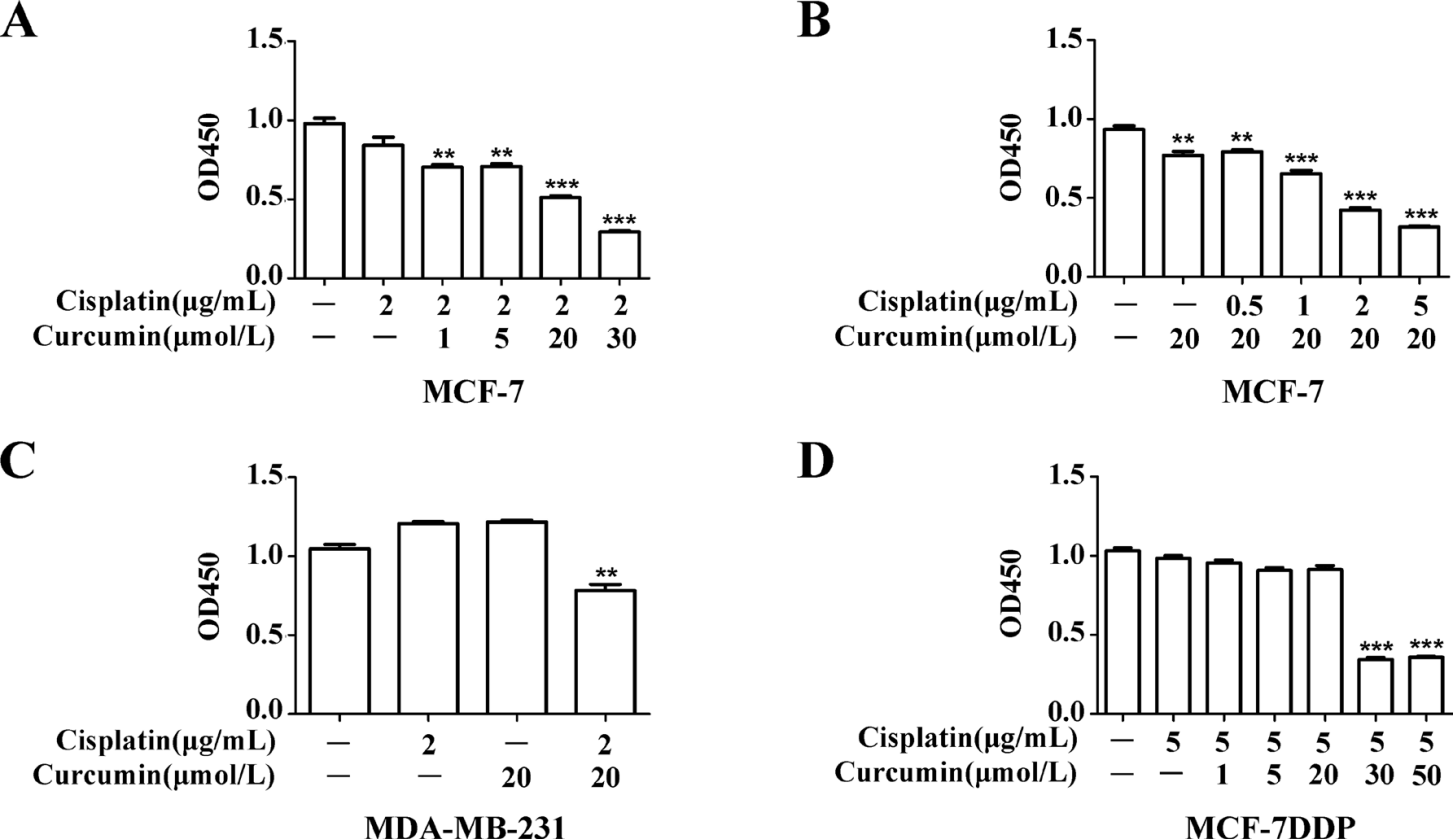
Curcumin’s effect on breast cancer cell sensitivity to cisplatin (**A**) MCF-7 cells were treated with 2 μg/mL cisplatin combined with increasing curcumin concentrations for 48 h, and cell proliferation was analyzed by CCK-8 assay. (**B**) MCF-7 cells were treated with 20 μmol/L curcumin and increasing cisplatin concentrations for 48 h, and cell proliferation was analyzed by CCK-8 assay. (**C**) MDA-MB-231 cells were treated with 2 μg/mL cisplatin and 20 μmol/L curcumin for 48 h, and cell proliferation was analyzed by CCK-8 assay. (**D**) MCF-7DDP cells were treated with 5 μg/mL cisplatin and increasing curcumin concentrations for 48 h, and cell proliferation was analyzed by CCK-8 assay. ^**^P < 0.01, ^***^P < 0.001.

MCF-7 cells were treated with 2 μg/mL cisplatin alone or combined with different concentrations of curcumin, and the resulting cell proliferation was analyzed (Figure 5A). Compared with the cells treated with 2 μg/mL cisplatin alone, the proliferation of cells treated with cisplatin in combination of ≥ 20 μmol/L curcumin was inhibited (*P* < 0.01). Compared with cells treated with 20 μmol/L curcumin alone (Figure 5B), the proliferation of cells treated with curcumin in combination with ≥ 2 μg/mL cisplatin was also inhibited (*P* < 0.01). In the MDA-MB-231 breast cancer cell line, the proliferation of cells treated with 20 μmol/L curcumin in combination with 2 μg/mL cisplatin was also inhibited compared with the cells treated with curcumin or cisplatin alone (Figure 5C, *P* < 0.01).

MCF-7DDP cells were treated with 5 μg/mL cisplatin alone or combined with different curcumin concentrations (Figure 5D). The proliferation of cells treated with cisplatin in combination with ≥ 30 μmol/L curcumin was inhibited (*P* < 0.001), compared with cells treated with cisplatin alone. These data suggest that, whether in wild-type breast cancer cells or in cisplatin-resistant breast cancer cells, the combination of cisplatin and curcumin can similarly enhance cell sensitivity to cisplatin.

**Figure 6:**
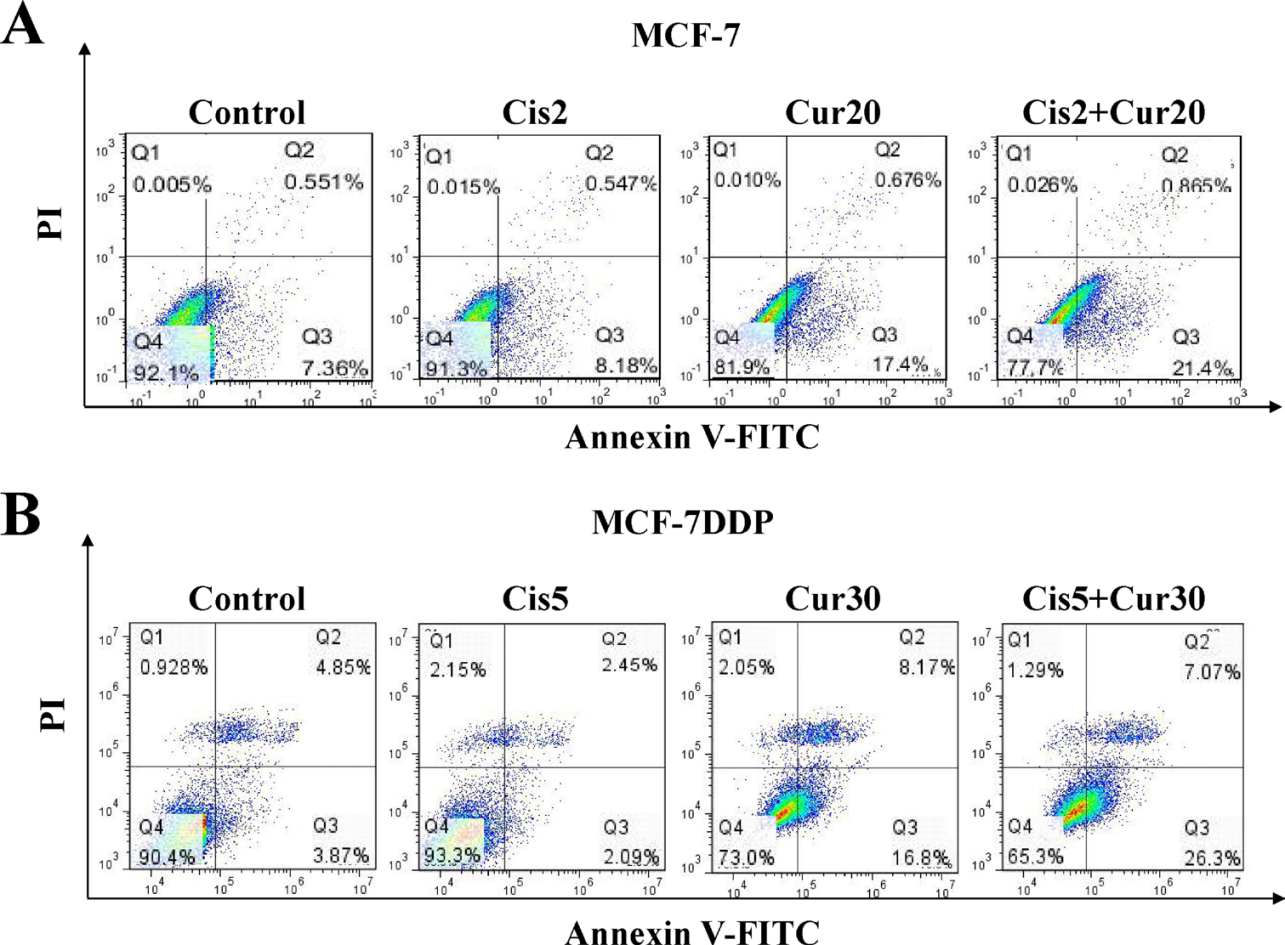
The combination of cisplatin and curcumin stimulates breast cancer cell apoptosis (**A**) MCF-7 cells were treated with 2 μg/mL cisplatin (Cis2) alone, or 20 μmol/L curcumin (Cur20) alone, or a combination of both for 48 h. They were then stained with annexin V-FITC/PI and analyzed by flow cytometry. (**B**) MCF-7DDP cells were treated with 5 μg/mL cisplatin (Cis5) alone, or 30 μmol/L curcumin (Cur30) alone, or combination of both for 48 h. Then the cells were stained with annexin V-FITC/PI and analyzed by flow cytometry.

Apoptosis of MCF-7 and MCF-7DDP cells treated with cisplatin alone or combined with curcumin was also observed. Both in MCF-7 cells treated with 2 μg/mL cisplatin and 20 μmol/L curcumin (Figure 6A) and in MCF-7DDP cells treated with 5 μg/mL cisplatin and 30 μmol/L curcumin (Figure 6B), the apoptosis in the combination treatment group was increased, compared with the single drug treatment group (*P* < 0.05). This suggests that the cisplatin and curcumin combination could improve breast cancer cell sensitivity to cisplatin.

**Figure 7:**
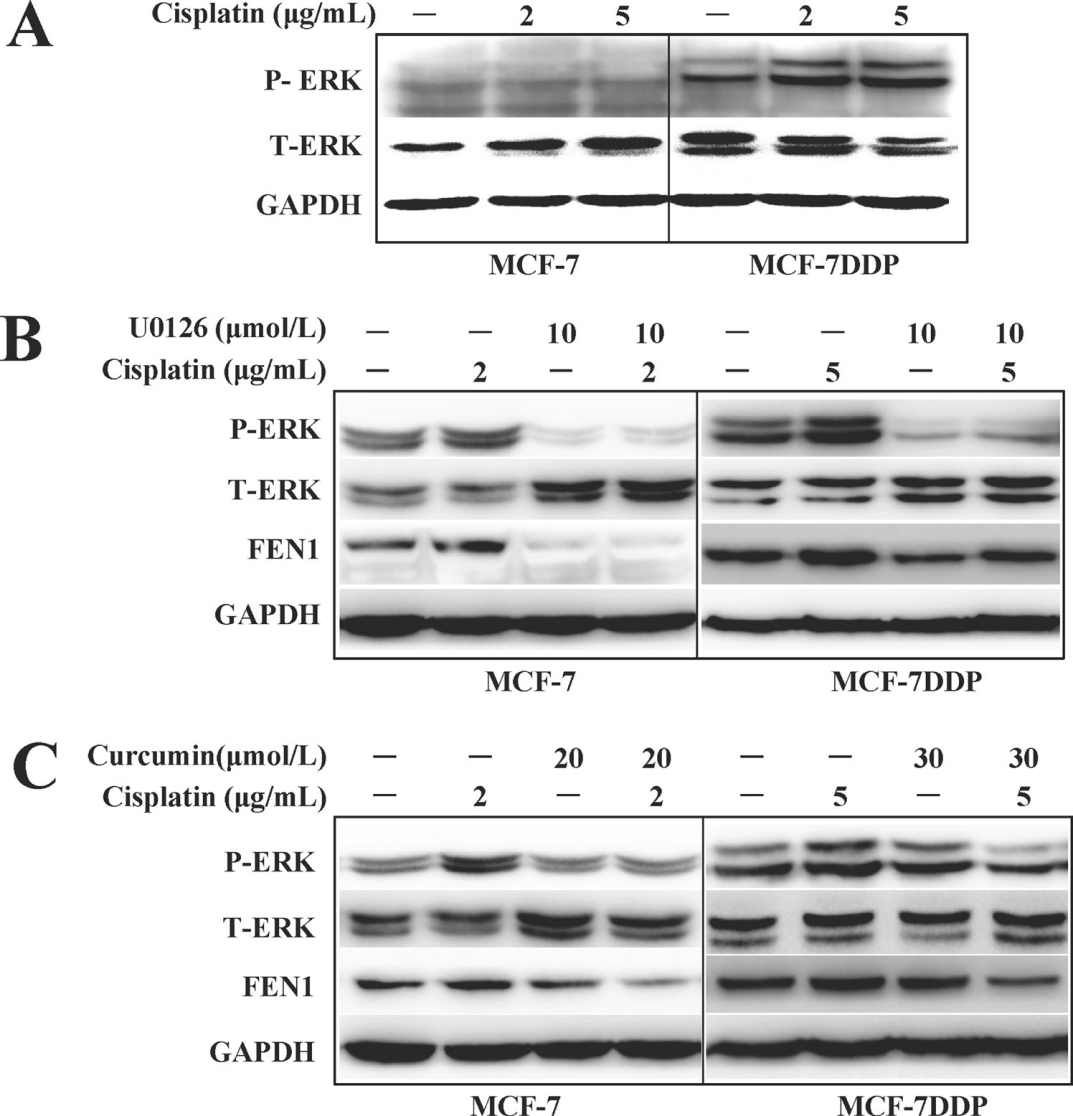
ERK phosphorylation changes and FEN1 expression in breast cancer cells treated with cisplatin, curcumin, U0126 alone, or their combination (**A**) MCF-7 or MCF-7DDP cells were treated with 2 μg/mL or 5 μg/mL cisplatin for 30 min respectively, and the ERK1/2 phosphorylation was detected by western blotting. (**B**) MCF-7 or MCF-7DDP cells were treated with or without 2 μg/mL or 5 μg/mL cisplatin combined with or without 10 μM/mL U0126 for 30 min, and the ERK1/2 phosphorylation was detected by western blotting. After the cells were subjected to treatment for another 24 h, the cell lysates were extracted for detection of FEN1 protein expression. (**C**) MCF-7 and MCF-7DDP cells were treated with or without 2 μg/mL or 5 μg/mL cisplatin combined with or without 20 μmol/L or 30 μmol/L curcumin for 30 min, and ERK1/2 phosphorylation and FEN1 protein expression were detected by western blotting.

The chemosensitizing effect of curcumin to cisplatin is closely related to curcumin-induced FEN1 down-regulation. In both MCF-7 and MCF-7DDP cells, FEN1 expression in the combination treatment group was decreased compared to the cisplatin only treatment group (Figure 7C). In addition, FEN1 overexpression in MCF-7 cells could block the chemosensitizing effect of 20 μmol/L curcumin to 2 μg/mL cisplatin, and silencing FEN1 expression in MCF-7DDP cells could increase the chemosensitizing effect of 20 μmol/L curcumin to 5 μg/mL cisplatin (Supplementary Figure 2). This suggests that the chemosensitizing effect of curcumin to cisplatin in breast cancer cells is achieved through FEN1 down-regulation.

### The inhibition of ERK phosphorylation is involved in the chemosensitizing effect of curcumin to cisplatin by targeting FEN1

We found that basal level ERK phosphorylation in MCF-7DDP cells was higher than in MCF-7 cells. The rapidly increased ERK phosphorylation could be induced by different concentrations of cisplatin treatment in both breast cancer cell lines, especially in MCF-7DDP cells because of their higher HER2 expression (Figure 7A). After pretreatment with the ERK inhibitor U0126, the cisplatin-induced ERK phosphorylation in MCF-7 and MCF-7DDP cells disappeared, and FEN1 expression was down-regulated (Figure 7B). This suggests that ERK phosphorylation may contribute to cisplatin-induced FEN1 overexpression in breast cancer cells.

Cisplatin increased NF-κB and ELK phosphorylation, which was accompanied by up-regulated FEN1 expression, but this could be attenuated by U0126 treatment (Supplementary Figure 3). These data indicate that cisplatin-induced phosphorylation of NF-κB and ELK may promote FEN1 up-regulation. When MCF-7 cells were treated with 2 μg/mL cisplatin alone or combined with 20 μmol/L of curcumin, and MCF-7DDP cells were treated with 5 μg/mL cisplatin alone or combined with 30 μmol/L of curcumin (Figure 7C), ERK phosphorylation and FEN1 expression levels were decreased in the combination treatment group compared with the cisplatin alone treatment group. These data suggested that curcumin enhances breast cancer cell sensitivity to cisplatin by down-regulating FEN1 expression.

### The combination therapy with cisplatin and curcumin significantly inhibited tumor growth and decreased FEN1 expression in a nude mouse xenograft model

**Figure 8:**
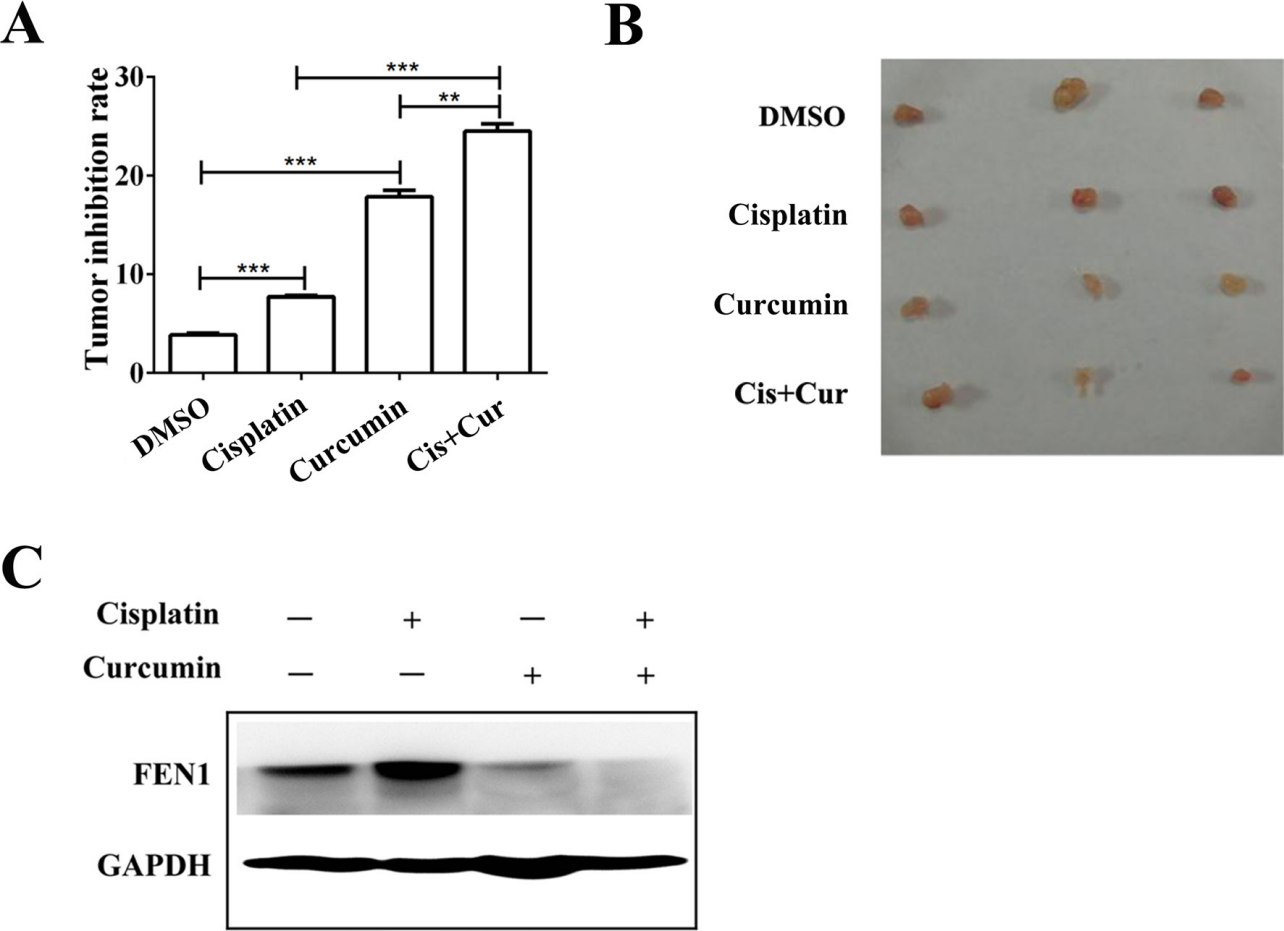
Curcumin enhances cisplatin antitumor effects through FEN1 down-regulation *in vivo* (**A**) Nude mice (*n* = 4) bearing subcutaneous tumors were treated intraperitoneally with DMSO, cisplatin, curcumin alone, or their combination, and the tumor volume was calculated as described in Materials and methods. ^**^P < 0.01, ^***^P < 0.001. (**B**) Representative images of tumor-bearing mice in each group. (**C**) FEN1 protein expression in each group. GAPDH served as the loading control.

MCF-7 human breast cancer xenografts in nude mice were used to evaluate the antitumor effect of cisplatin or curcumin treatment alone, or their combination, *in vivo*. Treatment with cisplatin or curcumin alone inhibited MCF-7 xenograft growth compared with the control. However, the combination of cisplatin and curcumin inhibited tumor growth more than either of the agents alone (*P* < 0.01, Figure 8A and 8B). Cisplatin treatment alone increased FEN1 expression in the xenograft tumors (*P* < 0.05). The combination of cisplatin and curcumin decreased FEN1 expression (Figure 8C).

## DISCUSSION

Cisplatin resistance inhibits the effectiveness of cisplatin treatment. The known resistance mechanisms focus on increased DNA repair, altered drug cellular accumulation, and increased drug cytosolic inactivation [17]. The DNA repair mechanism involved in cisplatin resistance mainly includes nucleotide-excision repair, mismatch repair, and base excision repair. Nucleotide excision repair is the most important mechanism involved in the DNA repair of cancer cells [18–20].

FEN1, a structure-specific nuclease, is an endonuclease that stimulates base excision repair. Once the coding region of FEN1 is changed, the genetic abnormality can lead to an abnormal increase or decrease of FEN1 content in the body, and functional deficiency of FEN1 can cause genomic instability and predisposition to cancer [4, 21]. FEN1 overexpression is correlated to cancer, and FEN1 is highly expressed in the cells of metastatic prostate cancer [22] , gastric cancer [23] , neuroblastomas [24], pancreatic cancer [25], lung cancer [26], and breast cancer [9, 10]. Our previous studies have shown that FEN1 down-regulation expression can effectively inhibit the proliferation of breast cancer cells overexpressing FEN1 [16]. Our present study found that cisplatin could induce FEN1 mRNA and protein up-regulation in a dose-dependent manner (Figures 2A, 3A ,7A, and Supplementary Figure 1). An RNAi-mediated down-regulation of FEN1 expression could enhance breast cancer cell sensitivity to cisplatin (Figure 3D and 3E). This suggests that FEN1 overexpression may promote cisplatin resistance, and that FEN1 could be a potential therapeutic target for the treatment of cisplatin resistance in breast cancer.

Curcumin can inhibit the growth of various cancer cells from different organs including breast cancer cells [27]. As a cancer chemosensitizing agent, curcumin can effectively eliminate resistance to many chemotherapy drugs, including cisplatin, mitomycin C and paclitaxel, in a wide variety of tumor cell types [28–32]. Because of its chemosensitizing effect, a combination of cisplatin with curcumin is proposed to improve the sensitivity of cisplatin. Although a combination of cisplatin with curcumin can reverse cisplatin resistance by promoting cell proliferation inhibition and apoptosis in lung cancer [30], Ke CS, et al [33] found that a combination therapy of curcumin with cisplatin could increase MCF-7 cell survival rate and exhibit an antagonizing effect. These dissimilar results suggest that the curcumin/cisplatin combination warrants further assessment.

Our study demonstrated that a combination of cisplatin and curcumin can effectively inhibit cell proliferation and increase apoptosis *in vitro* and *in vivo*, both in wild-type breast cancer cells or in cisplatin-resistant breast cancer cells (Figures 5A, 6A, and 8A). This combination effect benefits from curcumin-induced FEN1 down-regulation *in vitro* and *in vivo* (Figures 4C, 7C, 8B and Supplementary Figure 2). A combination of curcumin with cisplatin could enhance breast cancer cell sensitivity to cisplatin through down-regulation of FEN1 expression.

The possible mechanism of how curcumin could down-regulate FEN1 expression and increase the sensitivity of breast cancer cells to cisplatin was explored in this study. Our results showed that cisplatin-induced FEN1 expression can be eliminated by ERK inhibitor U0126 (Figure 7B), suggesting that ERK phosphorylation does contribute to cisplatin-induced FEN1 overexpression. After the breast cancer cells were subjected to a treatment with a combination of curcumin with cisplatin, both cisplatin sensitivity and FEN1 expression were decreased compared with cells treated with cisplatin alone. This suggests that curcumin can down-regulate FEN1 expression by reducing ERK phosphorylation levels, and then increase breast cancer cell sensitivity to cisplatin.

Though cisplatin-induced phosphorylation of NF-κB and ELK via ERK signaling were correlated with FEN1 up-regulation (Supplementary Figure 3), further study is needed to decide whether curcumin could down-regulate FEN1 expression through the specific transcription factors or epigenetic mechanisms such as acetylation and methylation.

**Figure 9:**
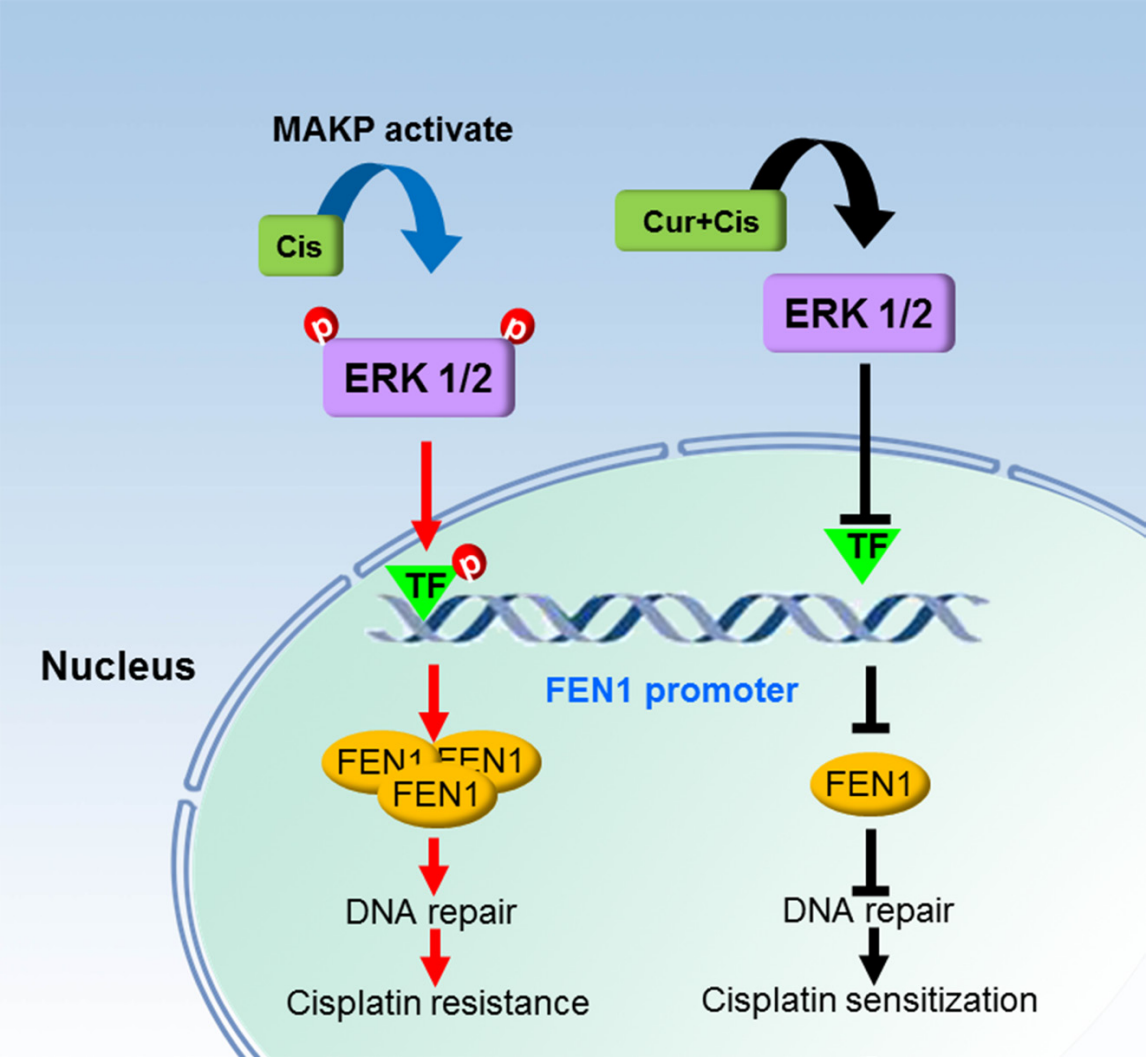
Schematic diagram of a mechanism by which FEN1 overexpression is associated with cisplatin resistance and chemosensitizing effect of curcumin in breast cancer cells Cisplatin treatment in breast cancer cells activates the ERK/MAPK pathway and cisplatin-activated ERK activates the related transcription factors, which bind to the FEN1 promoter. This eventually enhances FEN1 protein production, and FEN1 overexpression promotes cisplatin therapy resistance via increasing DNA repair ability. Curcumin treatment induces a decrease of ERK phosphorylation and inactivation of the given transcription factors, which cannot bind to the FEN1 promoter, and results in FEN1 down-regulation. Finally, curcumin enhances breast cancer cell sensitivity to cisplatin via attenuating DNA repair ability.

In conclusion, FEN1 overexpression stimulates cisplatin therapy resistance. Curcumin can enhance breast cancer cell sensitivity to cisplatin by down-regulating FEN1 expression, which is accomplished by decreasing curcumin-induced ERK phosphorylation. We depicted a schematic diagram illustrating how FEN1 overexpression is associated with cisplatin resistance and the chemosensitizing effect of curcumin in breast cancer cells (Figure 9). Our results provide new mechanisms for the research on cisplatin resistance and curcumin as a cisplatin-sensitizing agent in breast cancer cells. FEN1 could be a potential therapeutic target for the treatment of cisplatin resistance in breast cancer.

## MATERIALS AND METHODS

### Chemicals, antibodies, and plasmids

Curcumin, cisplatin, and U0126 were purchased from Sigma-Aldrich Corp. (St. Louis, MO, USA). Cell Counting Kit-8 (CCK-8) was from Dojindo (Shanghai, China). Mouse monoclonal anti-FEN1 antibody was purchased from Santa Cruz Biotechnology (Santa Cruz, CA, USA). The total and phosphorylated ERK1/2 (p-ERK1/2, Thr202/Tyr204) antibodies were purchased from Cell Signaling Technology (Boston, MA, USA). GAPDH antibody, HRP-conjugated goat anti-rabbit antibody, and HRP-conjugated goat anti-mouse antibody were purchased from Zhongshan Company (Beijing, China). Expression plasmid, pIRES, and expression plasmid for FEN1, pIRES-FEN1, were provided by Dr. Binghui Shen in the Department of Radiation Biology, Beckman Research Institute of City Hope, Duarte, California, USA.

### Cell culture

Human breast cancer cell lines MCF-7, BT-474, and MDA-MD-231 were purchased from American Type Culture Collection (ATCC), and characterized by DNA profiling. Cells were cultured in high-glucose Dulbecco’s modified Eagle’s medium (DMEM) with 10% fetal bovine serum (FBS), 1% non-essential amino acids (NEAAs), streptomycin (100 μg/mL), and penicillin (100 U/mL) in a humidified atmosphere of 5% CO2 at 37°C. MCF-7 cells with high cisplatin concentrations were used to create 5 μg/mL cisplatin-resistant strains (MCF-7DDP). Cisplatin was added twice a week after reseeding. Every two months, cell survival was analyzed by MTT assay. The IC50 value of cisplatin against MCF-7 and MCF-7DDP were 4 μg/mL and 15 μg/mL, respectively. MCF-7DDP cells were four times as resistant to the cytotoxic effect of cisplatin as compared with the initial MCF-7 cells.

### Cell proliferation assay

Cells were seeded in 96-well plates with 5 × 10^3^ cells/well, followed by treatment with DMSO (vehicle) or increasing cisplatin concentrations (0, 1.25, 2.5, 5, 10, and 20 μg/mL) and/or curcumin (0, 1, 5, 20, 30, 50, and 100 μmol/L) for 48 h. Cells were incubated in 10 μl CCK-8 reagent for 1 h, and the OD value was measured at 450 nm according to the manufacturer’s instructions.

### Flow cytometry

Breast cancer cells were incubated with annexin V-FITC and PI according to the manufacturer’s instructions (BD, 561012), and then the apoptosis was analyzed by flow cytometry.

### Establishment of FEN1 stable expression cell lines

MCF-7 cells were transfected with either pIRES or pIRES-FEN1, using Lipofectamin 2000 (Invitrogen, Grand Island, NY, USA). At 72 h after transfection, G418 was then added to the transfected cells with an increasing concentration up to 500 μg/mL over a 1-month period. MCF-7 cells stably overexpressing FEN1 were identified by western blot, then used for the experiments.

### RNA interference

The short interfering RNA (siRNA) strand oligomers specific for FEN1 and its negative control siRNA (NC siRNA) were synthesized according to the reference [34]. Cells were plated in 6-well plates or 48-well plates, and incubated for 24 h. Next, cells were transfected with siRNA using the Lipofectamin 2000 in accordance with the manufacturer’s instructions. At 6 h after transfection, cells treated with or without 5 μg/mL cisplatin for 48 h, then harvested and used for western blot analysis or cell proliferation assays.

### Western blot analysis

Cells were seeded into 6-well plates with 0.5 × 10^6^ cells/well, followed by a treatment with various concentrations of cisplatin and curcumin for 30 min or 24 h. Isolation of cell extracts and western blot analysis were described previously [16] The cells were harvested and the protein concentrations were measured by BCA protein assay kit. Aliquots containing 50 μg of protein were resolved by 10% SDS-PAGE, followed by electrotransfer to a PVDF membrane. Immuno-detection was carried out using FEN1, p-ERK1/2, total-ERK antibody, or GAPDH antibody. GAPDH was used as a control for equal loading and transfer. Quantitative densitometry of the immuno-images was performed using the Model GS-700 Imaging Densitometer with Molecular Analyst Software (Bio-Rad Laboratories, Hercules, CA, USA), and expressed as the ratios to the density of GAPDH bands.

### Mouse tumor xenografts

Five-week-old female nude mice were purchased from the Laboratory Animal Center of China (Shanghai, China) and fed a sterilized mouse diet and water. The nude mice (*n* = 4) were anesthetized through isoflurane inhalation, and 1 × 10^7^ MCF-7 cells suspending in 0.1 ml PBS was injected subcutaneously into the right axillary of each mouse. After 14 days, the mice were randomly assigned to four groups (*n* = 3/group), and either treated with DMSO (control), curcumin (100 μg/kg), cisplatin (2 mg/kg), or curcumin plus cisplatin by intraperitoneal injections every other day for 2 weeks, respectively. The length and width of the xenograft tumors were monitored before and after treatment, and their volumes were estimated using the following formula: volume = width^2^ × length × 1/2. The mice were sacrificed and tumors were harvested for FEN1 expression analysis.

### Statistical analysis

Data are the mean ± SD obtained from at least three independent experiments. Statistical comparisons between groups were performed by one-way analysis of variance (ANOVA), followed by student’s *t*-test. *P* < 0.05 was considered significant.

## SUPPLEMENTARY MATERIALS FIGURES


