# Supplementary material for: Curcumin increases breast cancer cell sensitivity to cisplatin by decreasing FEN1 expression
Source: Oncotarget. 2018 Jan 10;9(13):11268–78. doi: 10.18632/oncotarget.24109 (PMC5834274; doi:10.18632/oncotarget.24109)
Supplement: Supplementary file 1 [file oncotarget-09-11268-s001.pdf]

Curcumin increases breast cancer cell sensitivity to cisplatin by decreasing FEN1 expression

SUPPLEMENTARY MATERIALS

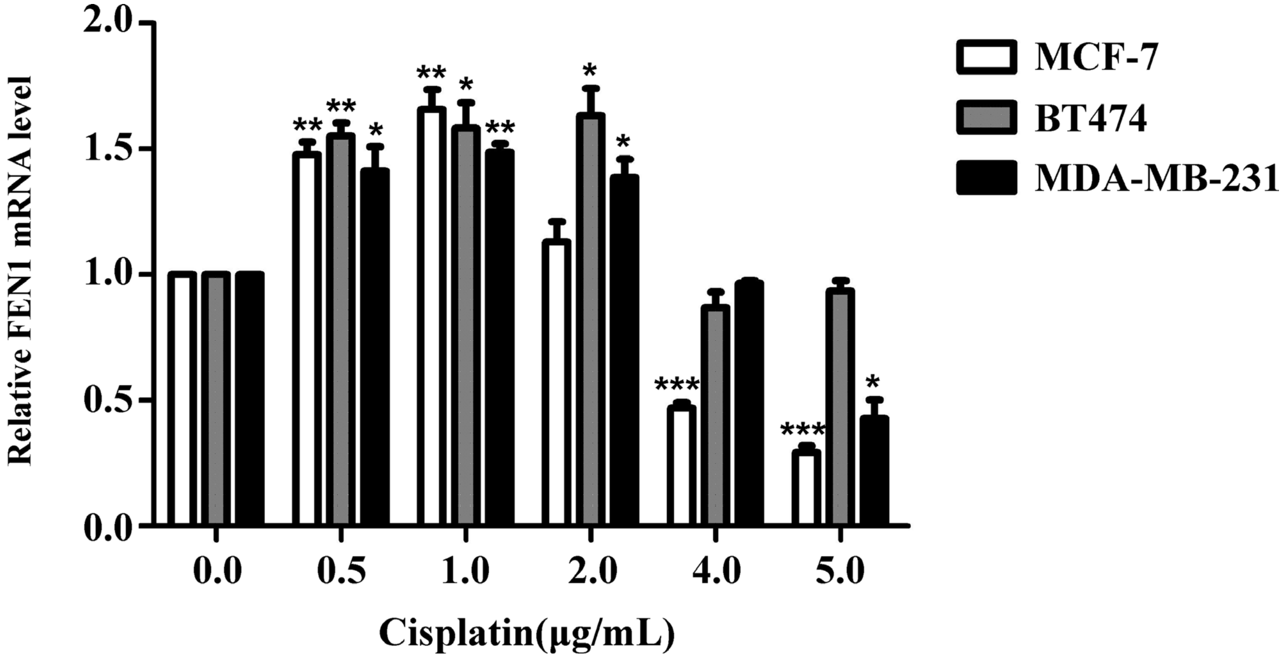

Supplementary Figure 1: Cisplatin induced FEN1 mRNA up-regulation in breast cancer cells. MCF-7, BT-474, and MDA-MB-231 cells were treated with increasing cisplatin concentrations for 24 h. FEN1 mRNA level was examined by qPCR, and the data was expressed as the fold change over the control. $^{*}P < 0.05$, $^{**}P < 0.01$, $^{***}P < 0.001$.

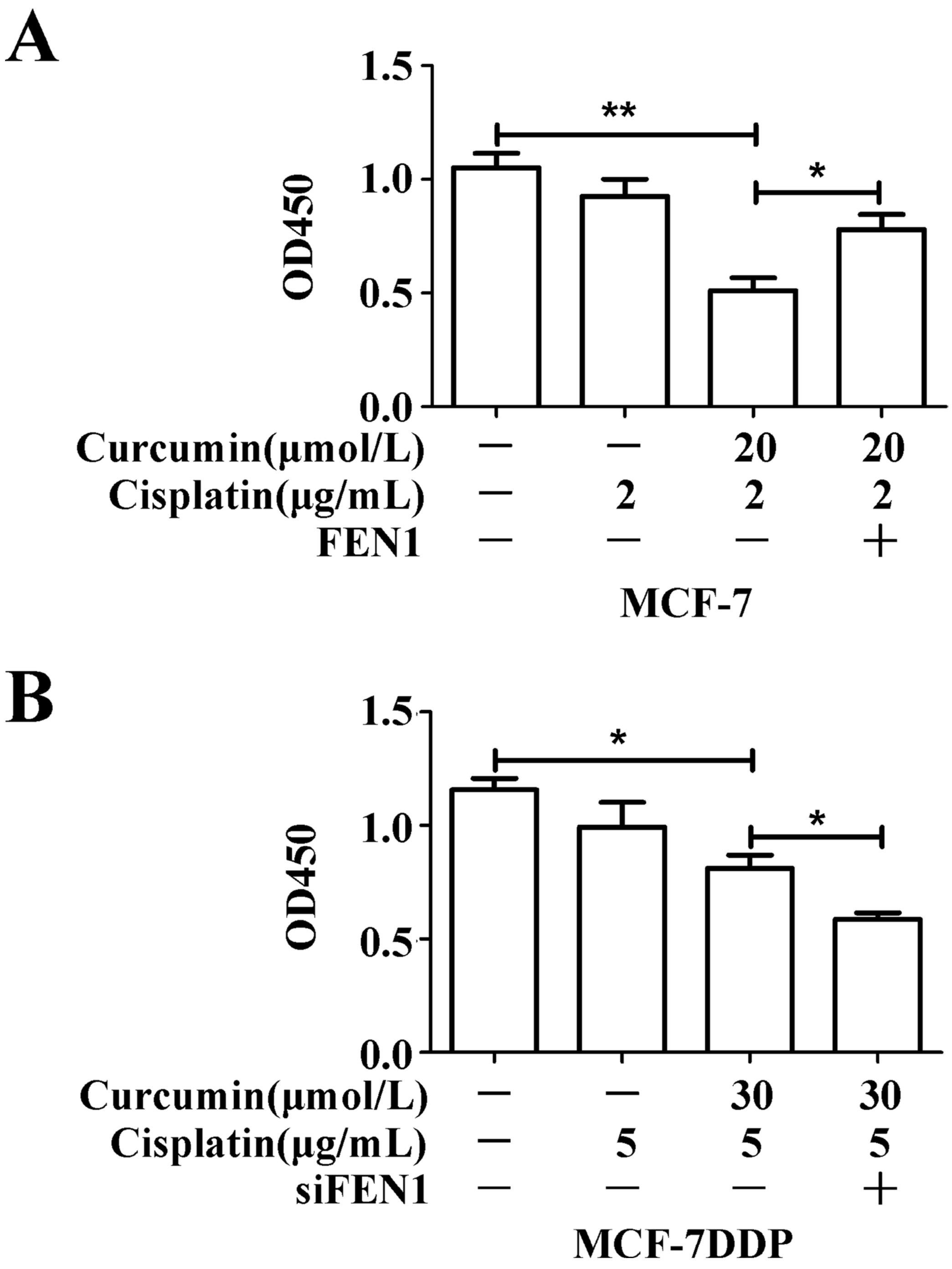

Supplementary Figure 2: The chemosensitizing effect of curcumin to cisplatin in breast cancer cells is mediated through down-regulation of FEN1 expression. (**A**) MCF-7 cells stably overexpressing FEN1 and control cells were treated with or without 2 μg/mL cisplatin combined with 20 μmol/L curcumin for 48 h. Cell proliferation was analyzed by CCK-8 assay. $^{*}P < 0.05$. (**B**) MCF-7DDP cells were transfected with FEN1 siRNA, and its negative control siRNA were treated with or without 5 μg/mL cisplatin combined with 20 μmol/L curcumin for 48 h. Cell proliferation was analyzed by CCK-8 assay. $^{*}P < 0.05$.

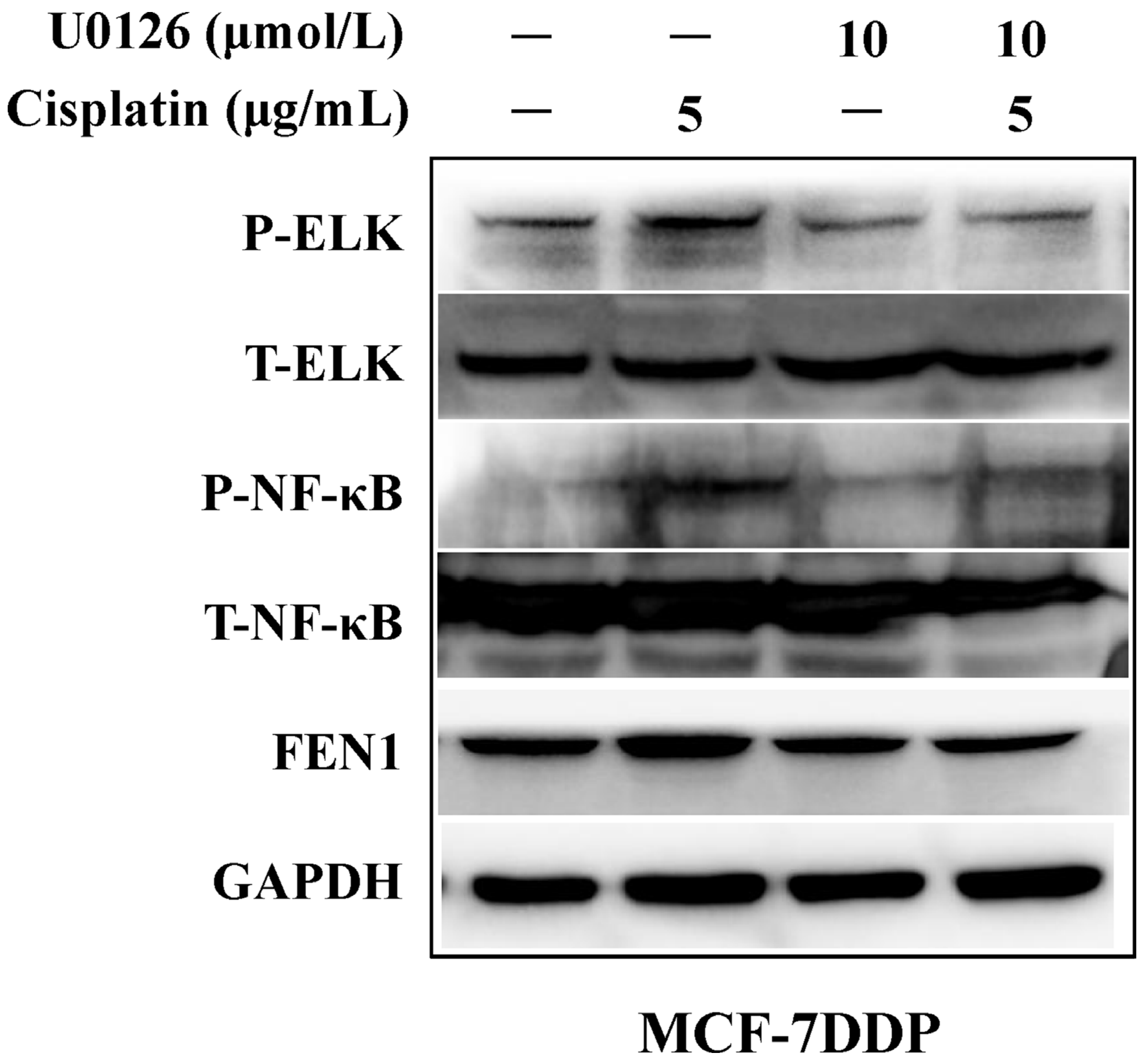

Supplementary Figure 3: The correlation between the phosphorylation changes of NF-κB or ELK and the changes of FEN expression in MCF-7/DDP cells. MCF-7/DDP cells treated with or without 5 µg/mL cisplatin combined with or without 10 µM/mL U0126 for 30 min. ELK, NF-κB, and ERK1/2 phosphorylation and FEN1 protein were detected by western blotting as described in Figure 7B.
